# Compressive Behavior of Fiber-Reinforced Concrete with End-Hooked Steel Fibers

**DOI:** 10.3390/ma8041442

**Published:** 2015-03-27

**Authors:** Seong-Cheol Lee, Joung-Hwan Oh, Jae-Yeol Cho

**Affiliations:** 1 Department of NPP Engineering, KEPCO International Nuclear Graduate School, 658-91 Haemaji-ro, Seosaeng-myeon, Uljugun, Ulsan 689-882, Korea; E-Mail: sclee@kings.ac.kr; 2 Office of Offshore Wind Power, Korea Institute of Energy Technology Evaluation and Planning, Seoul 135-502, Korea; E-Mail: ffung45@ketep.re.kr; 3 Department of Civil and Environmental Engineering, Seoul National University, Seoul 151-744, Korea

**Keywords:** steel fiber, end-hooked fiber, SFRC, compression, elastic modulus, strain at the compressive strength

## Abstract

In this paper, the compressive behavior of fiber-reinforced concrete with end-hooked steel fibers has been investigated through a uniaxial compression test in which the variables were concrete compressive strength, fiber volumetric ratio, and fiber aspect ratio (length to diameter). In order to minimize the effect of specimen size on fiber distribution, 48 cylinder specimens 150 mm in diameter and 300 mm in height were prepared and then subjected to uniaxial compression. From the test results, it was shown that steel fiber-reinforced concrete (SFRC) specimens exhibited ductile behavior after reaching their compressive strength. It was also shown that the strain at the compressive strength generally increased along with an increase in the fiber volumetric ratio and fiber aspect ratio, while the elastic modulus decreased. With consideration for the effect of steel fibers, a model for the stress–strain relationship of SFRC under compression is proposed here. Simple formulae to predict the strain at the compressive strength and the elastic modulus of SFRC were developed as well. The proposed model and formulae will be useful for realistic predictions of the structural behavior of SFRC members or structures.

## 1. Introduction

Recently, as the demand for high strength concrete has increased, the structural behavior of reinforced concrete has become more brittle. In order to reduce this side effect, steel fiber-reinforced concrete (SFRC) has arisen as a viable method to attain ductility not only during post-cracking behavior under tension, but also during post-peak softening behavior under compression. For the last several decades, use of steel fibers has been limited to tunnel lining or crack control in concrete slabs as a non-structural material. Development of a rational compressive model for SFRC has been of importance since many researchers [1,2,3,4,5,6,7,8,9,10] have continuously conducted experimental and analytical research to exploit SFRC as a structural material.

Several researchers [11,12,13,14,15,16,17] have investigated the effect of steel fibers on the compressive behavior of SFRC for design purposes. Table 1 summarizes models of the compressive behavior of SFRC, which several independent research groups have developed based on their own test results. As shown in the table, in order to predict the compressive stress-strain response of SFRC, the measured compressive strength of SFRC is required in some models [11,15], while in the other models [12,13,14], the compressive strength of SFRC is derived from the compressive strength of plain concrete without fibers. In the models summarized in the table, the coefficient *β*, introduced first by Carreria and Chu [18] for normal concrete without fibers, has been modified to reflect the effect of steel fibers on compressive behavior. However, since the models proposed by Ezeldin and Balaguru [11], Someh and Saeki [13], and Nataraja *et al.* [15] were developed based on test results with crimped steel fibers, the appropriateness of the models has become questionable for concrete with end-hooked steel fibers.

The model presented by Hsu and Hsu [12] considers only several specific fiber volumetric ratios (0.50%, 0.75% and 1.00%), so its practical application is quite limited. In addition, in the experiments conducted by some research groups [12,13,14], only one fiber aspect ratio (length to diameter) was considered, so it is questionable whether the models proposed by those research groups reasonably represent the effect of the aspect ratio of steel fibers on compressive behavior. Moreover, some specimens [11,12] tested in the development of previous models were relatively small in comparison with fiber length, so the observed compressive behavior of SFRC could differ from that in real structures that are relatively large because fiber distribution can be significantly affected by the boundary surfaces of small specimens [19,20]. Some models [11,15] require data on the compressive strength of plain concrete without fibers in the same mix proportion as SFRC, but it is more practical to use the actual compressive strength of SFRC in the model. Consequently, a more reasonable model to represent the compressive behavior of SFRC should be developed from a more extensive experimental program using large specimens and various steel fiber configurations.

**Table 1:** Models for the compressive behavior of fiber reinforced concrete.

Researchers	Models
^†^Ezeldin and Balaguru [11]	$f_{c}={f_{c}}^{\prime }\frac{\beta \left(\frac{\varepsilon }{\varepsilon_{0}}\right)}{\beta -1+{\left(\frac{\varepsilon }{\varepsilon_{0}}\right)}^{\beta }}$ where, ${f_{c}}^{\prime }={f_{cp}}^{\prime }+11.232RI$;$\beta =1.093+0.2429RI^{-0.926}$; $E_{c}=E_{cp}+9936RI$; $\varepsilon_{0}=\varepsilon_{0p}+1427\times 10^{-6}RI$; ${f_{cp}}^{\prime }$, $\varepsilon_{op}$, and $E_{cp}$ are the compressive strength, corresponding strain, and elastic modulus of plain concrete, respectively.
Hsu and Hsu [12]	$f_{c}={f_{c}}^{\prime }\frac{n\beta \left(\frac{\varepsilon }{\varepsilon_{0}}\right)}{n\beta -1+{\left(\frac{\varepsilon }{\varepsilon_{0}}\right)}^{n\beta }}$ where for $0\leq \frac{\varepsilon }{\varepsilon_{0}}\leq \frac{\varepsilon_{d}}{\varepsilon_{0}}$ $f_{c}=0.6{f_{c}}^{\prime }\exp\left[-0.7{\left(\frac{\varepsilon }{\varepsilon_{0}}-\frac{\varepsilon_{d}}{\varepsilon_{0}}\right)}^{0.8}\right]$for $\frac{\varepsilon_{d}}{\varepsilon_{0}}\leq \frac{\varepsilon }{\varepsilon_{0}}$ where, $\varepsilon_{d}$ is the strain at $0.6{f_{c}}^{\prime }$ in the descending; ${f_{c}}^{\prime }$ is the measured compressive strength; $\beta ={\left(\frac{{f_{c}}^{\prime }}{11.838{\left(100V_{f}\right)}^{3}+58.612}\right)}^{3}-26V_{f}+2.742$; $E_{i}=a_{2}{f_{c}}^{\prime }+C_{2}$; $\varepsilon_{0}=a_{1}{f_{c}}^{\prime }+C_{1}$ where, $a_{1}$, $a_{2}$, $C_{1}$, and $C_{2}$ are constant.
^†^Someh and Saeki [13]	$f_{c}={f_{c}}^{\prime }\frac{\beta \left(\frac{\varepsilon }{\varepsilon_{0}}\right)}{\beta -1+{\left(\frac{\varepsilon }{\varepsilon_{0}}\right)}^{\beta }}$ where, ${f_{c}}^{\prime }$ is the measured compressive strength; $\beta =1.032{\left[{f_{c}}^{\prime }\left(1+RI\right)\right]}^{0.113}$; and $\varepsilon_{0}=0.00184{f_{c}}^{\prime 0.147}$.
^†^Mansur *et al.* [14]	$f_{c}={f_{c}}^{\prime }\frac{\beta \left(\frac{\varepsilon }{\varepsilon_{0}}\right)}{\beta -1+{\left(\frac{\varepsilon }{\varepsilon_{0}}\right)}^{\beta }}$ for $0\leq \frac{\varepsilon }{\varepsilon_{0}}\leq 1$ $f_{c}={f_{c}}^{\prime }\frac{k_{1}\beta \left(\frac{\varepsilon }{\varepsilon_{0}}\right)}{k_{1}\beta -1+{\left(\frac{\varepsilon }{\varepsilon_{0}}\right)}^{k_{2}\beta }}$ for $1\leq \frac{\varepsilon }{\varepsilon_{0}}$ where, ${f_{c}}^{\prime }$ is the measured compressive strength; $\beta =1/\left[1-\left({f_{c}}^{\prime }/\varepsilon_{0}E_{i}\right)\right]$; $E_{i}=\left(10300-40000V_{f}\right){f_{c}}^{\prime 1/3}$; $\varepsilon_{0}=\left(0.00050+0.000072RI\right){f_{c}}^{\prime 0.35}$.
^†^Nataraja *et al.* [15]	$f_{c}={f_{c}}^{\prime }\frac{\beta \left(\frac{\varepsilon }{\varepsilon_{0}}\right)}{\beta -1+{\left(\frac{\varepsilon }{\varepsilon_{0}}\right)}^{\beta }}$ where, ${f_{c}}^{\prime }={f_{cp}}^{\prime }+6.9133RI$; $\beta =0.5811+0.8155RI^{-0.7406}$; $\varepsilon_{0}=\varepsilon_{0p}+0.00192RI$; ${f_{cp}}^{\prime }$ and $\varepsilon_{op}$ are the compressive strength of plain concrete and corresponding strain, respectively.

Note: ^†^ $RI=V_{f}l_{f}/d_{f}$ where $V_{f}$ is a fiber volumetric ratio.

## 2. Test Program for the Compressive Behavior of SFRC

**Figure 1:**
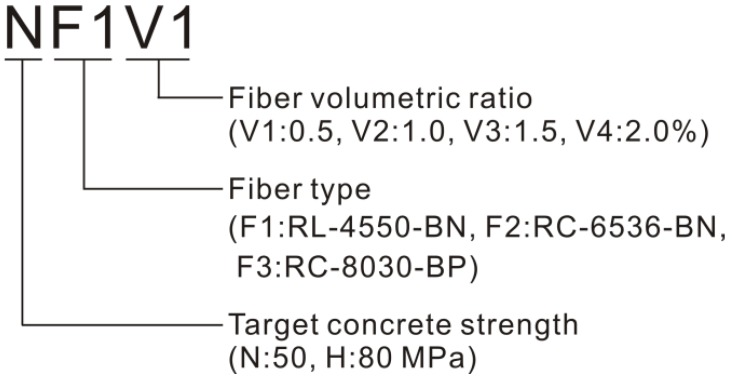
Test variables.

In this study, the compression test with *ϕ* 150 mm by 300 mm cylindrical specimens was conducted to investigate the compressive behavior of SFRC. The variables were concrete compressive strength ($f_{c}^{\prime }$), fiber volumetric ratio ($V_{f}$), and fiber aspect ratio ($l_{f}/d_{f}$). Four fiber volumetric ratios ranging from 0.5% to 2.0% were considered, and end-hooked steel fibers with three different aspect ratios (47.6%, 63.6%, and 78.9%) were used. In order to investigate the effect of concrete compressive strength, two compressive strengths of 50 and 80 MPa were targeted for N and H series, respectively. In the test for H series, only steel fibers 30 mm in length and 0.38 mm in diameter were used since it was inferred that those fibers would be the most effective for optimizing ductile performance during post-peak compressive behavior, as shown in a previous test of the tensile behavior of SFRC [21]. Specifications of the steel fibers are presented in Table 2, and the notation for the test variables is illustrated in Figure 1.

**Table 2:** Properties of steel fibers.

Type	Length *l_f_* [mm]	Diameter *d_f_* [mm]	Tensile strength *σ_fu_* [MPa]	Aspect ratio *l_f_*/*d_f_*
F1: RL-4550-BN *	50	1.05	1000	47.6
F2: RC-6535-BN *	35	0.55	1100	63.6
F3: RC-8030-BP *	30	0.38	2300	78.9

Note: * Fiber model names are given by the manufacturer, Bekaert.

The concrete mix proportion of normal concrete without fibers is difficult to apply for SFRC since steel fibers reduce the workability for a given mixture design. Therefore, finer aggregate and cement should replace a portion of the coarse aggregate to guarantee both workability and target compressive strength. In this study, the concrete mixture design presented in Susetyo [22] was modified as presented in Table 3. The maximum aggregate size was 13 mm and Type I cement was used. Before mixing the concrete, the coarse aggregate was dried for one day after being submerged in water so that surface saturation could be achieved when the concrete was mixed. The mixing procedure presented in Susetyo [22] was followed, and cylindrical specimens were cast in two phases. After each half casting, the specimens were vibrated for 5 seconds on a vibrating table at 5 Hz.

**Table 3:** Mix proportions.

Concrete strength	Water to binder ratio	Water [kg/m^3^]	Cement [kg/m^3^]	Silica fume [kg/m^3^]	Sand [kg/m^3^]	Coarse aggregate [kg/m^3^]	Super-plasticizer [kg/m^3^]
Normal strength (N)	0.35	200	572	-	798	627	1.430
High strength (H)	0.25	200	737	64	667	569	6.008

Three cylinders per test series were cast, which resulted in 48 specimens for 16 test series. The specimens were demolded one day after casting and then cured at 20 °C and 70% relative humidity until the age of 28 days, when the compression tests were conducted. To prevent the specimens from being subjected to possible eccentric loading, both the top and bottom surfaces of all specimens were ground. Two linear variable differential transformers (LVDTs) with a reference length of 150 mm were mounted on the circumferential side of the specimens so that the average longitudinal strain could be simultaneously measured during the compression test. The specimens were subjected to uniaxial compression under a stroke control of 0.4 mm/min.

## 3. Test Results and Investigations

### 3.1. Pre-Peak Compressive Behavior

The pre-peak compressive behavior of SFRC can be represented by several characteristics such as concrete compressive strength, compressive strain at the peak, and elastic modulus. These characteristics, as measured from the compression test, are summarized in Table 4. The test results were obtained from the average for three specimens per test series.

**Figure 2:**
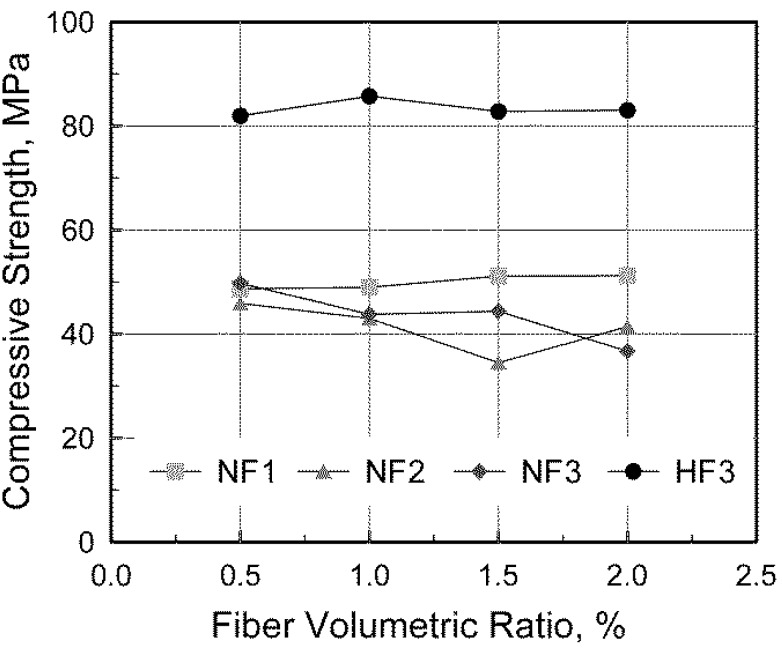
Measured compressive strength.

The effect of the fiber volumetric ratio on compressive strength can be seen in Figure 2. As shown in the figure, the compressive strength increased slightly with increasing fiber volumetric ratio in the NF1 series, while it decreased in the NF3 series. This inconsistency has been noted by other researchers as well. Ezeldin and Balaguru [11] reported that the compressive strength increased with increasing steel fiber volume due to the transverse confinement effect of the steel fibers, which restrained the lateral expansion of SFRC specimens. On the other hand, Hsu and Hsu [12] reported that steel fibers did not contribute to an increase in the compressive strength since more voids could be produced in SFRC because of its low workability. These effects of steel fibers can also be explained with the measured slumps presented in Table 4. A large slump with good workability was observed in the NF1 series, in which relatively long steel fibers with a small aspect ratio were mixed, resulting in fewer steel fibers per unit of concrete volume. On the other hand, when NF3 series with shorter steel fibers with a large aspect ratio were mixed, resulting in a large number of steel fibers per unit concrete volume, a small slump was observed. It can be concluded, therefore, that the compressive strength of SFRC can be affected by the fiber aspect ratio as well as the fiber volumetric ratio.

**Table 4:** Test results.

Specimen.	Target strength [MPa]	$l_{f}/d_{f}$	$V_{f}$ [%]	${f_{c}}^{\prime }$ [MPa]	$\varepsilon_{0}$ [με]	$E_{c}$ [MPa]	Slump [mm]
NF1V1	50	45	0.5	48.7	3,137	25,406	146
NF1V2			1.0	49.0	3,047	25,781	154
NF1V3			1.5	51.1	3,190	25,187	129
NF1V4			2.0	51.2	3,244	26,091	111
NF2V1		65	0.5	45.9	2,701	29,987	113
NF2V2			1.0	43.0	3,150	22,812	135
NF2V3			1.5	34.5	2,289	26,107	141
NF2V4			2.0	41.4	3,187	21,758	83
NF3V1		80	0.5	49.8	3,078	24,373	111
NF3V2			1.0	43.8	3,100	26,661	114
NF3V3			1.5	44.4	3,422	20,640	83
NF3V4			2.0	36.7	3,499	22,880	39
HF3V1	80	80	0.5	81.9	3,191	31,328	225
HF3V2			1.0	85.7	3,424	33,581	193
HF3V3			1.5	82.8	3,288	33,855	164
HF3V4			2.0	83.0	3,922	28,246	118

The effect of the concrete mix proportion was also investigated by comparing NF3 and HF3 series, which contain the same fibers. As seen in Table 4 and Figure 2, in the NF3 series, the compressive strength generally decreased with an increase in the fiber volumetric ratio. In the HF3 series, on the other hand, the effect of the fiber volumetric ratio on the compressive strength was not obvious since good workability with a large slump was obtained by adopting a different mixture design, *i.e.*, silica fume was added to achieve higher compressive strength in the H series. Aside from NF2V3 and NF3V4 which exhibited the compressive strengths scattered from the others, it can be seen that the compressive strength of SFRC is not significantly affected by the fiber volumetric ratio.

**Figure 3:**
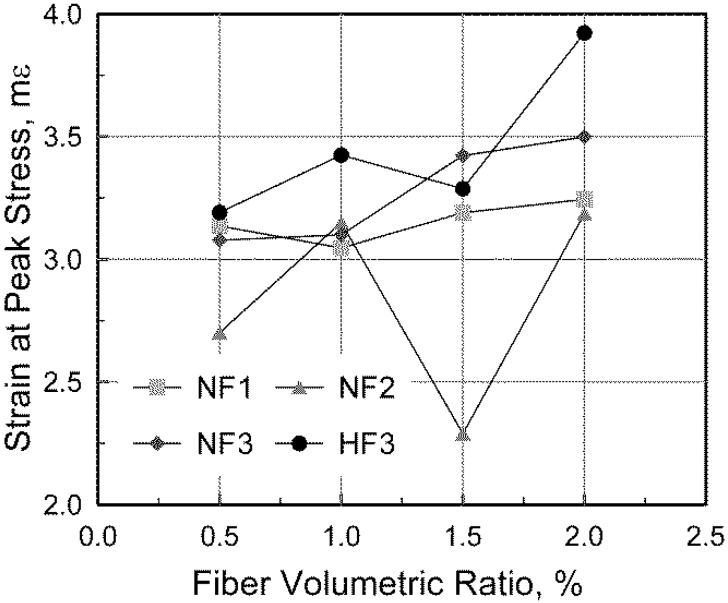
Measured compressive strain at the compressive strength.

Figure 3 shows the effect of the fiber volumetric ratio on the strain at the peak stress. As presented in the figure, in NF3 and HF3 series, in which shorter steel fibers with a high aspect ratio were mixed, the strain corresponding to the compressive strength significantly increased with an increase in the fiber volumetric ratio. This tendency was not clearly observed in the NF1 series, in which long steel fibers with a low aspect ratio were mixed. On the other hand, the other members except NF2V3 exhibited the general tendency regarding the effect of the fiber volumetric ratio. The test results indicate that the strain at the compressive strength is affected by both the fiber volumetric ratio and fiber aspect ratio, which correspond to the number of fibers per unit concrete volume.

From the measured compressive stress-strain responses, the elastic modulus was evaluated according to ASTM C469 [23] as follows:

(1) $$E_{c}=\frac{f_{c2}-f_{c1}}{\varepsilon_{c2}-\varepsilon_{c1}}$$

where $f_{c1}$ and $f_{c2}$ are the compressive stresses corresponding to $\varepsilon_{c1}=50\times 10^{-6}$ and $\varepsilon_{c2}$ at $0.4f_{c}^{\prime }$, respectively; and $f_{c}^{\prime }$ is the measured compressive strength.

**Figure 4:**
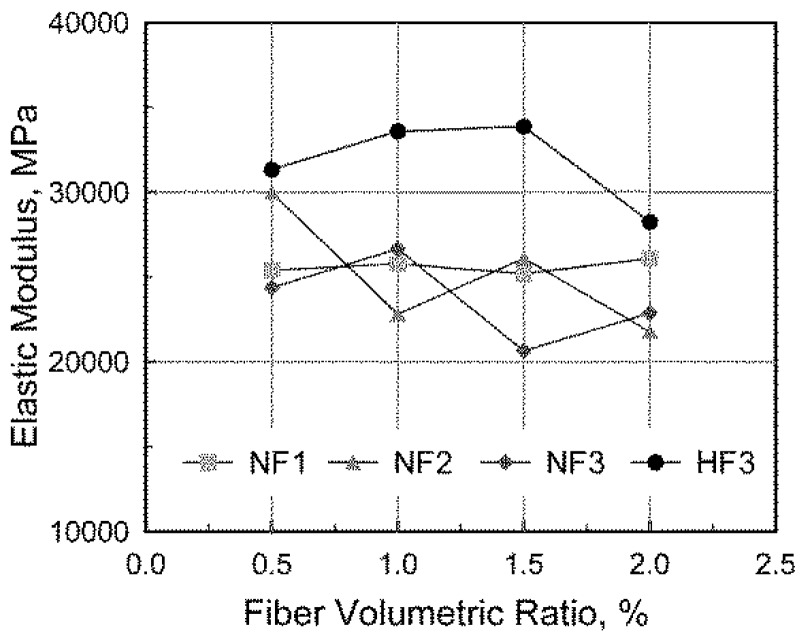
Measured elastic modulus.

The evaluated elastic modulus is presented in Figure 4. As shown in the figure, the elastic modulus in the NF2 and NF3 series generally decreased with an increase in the fiber volumetric ratio, while the elastic modulus in the NF1 series remained almost constant regardless of the fiber volumetric ratio. In the HF3 series, however, since fewer voids existed in the specimens because of the relatively high slump due to the different mixture design, it was not obvious that the fiber volumetric ratio had a significant effect on the elastic modulus when the fiber volumetric ratio was not greater than 1.5%.

### 3.2. Post-Peak Compressive Behavior

**Figure 5:**
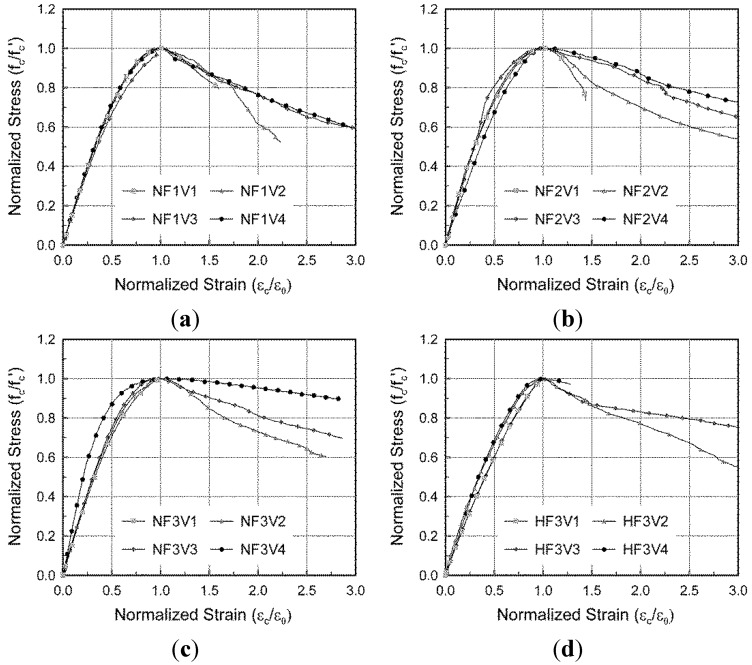
Effect of fiber volumetric ratio on the stress-strain response under compression. (**a**) NF1 series; (**b**) NF2 series; (**c**) NF3 series; (**d**) HF3 series.

**Figure 6:**
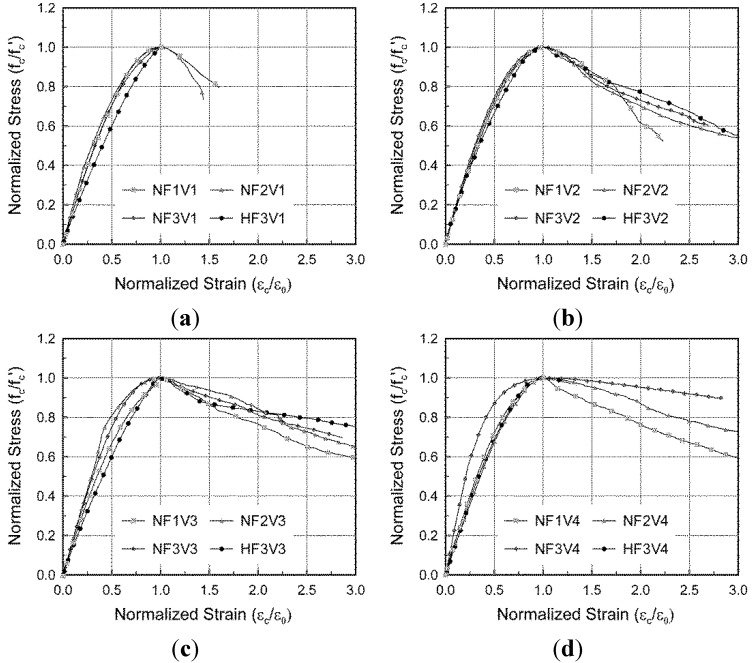
Effect of fiber aspect ratio on the stress-strain response under compression. (**a**) NF1 series; (**b**) NF2 series; (**c**) NF3 series; (**d**) HF3 series.

While normal concrete without steel fibers shows a drastic decrease in stress after experiencing compressive strength, it is well known that SFRC can exhibit ductile behavior even after reaching its compressive strength because of the transverse confinement effect of the steel fibers [11,12,13,14,15]. This ductile behavior was also indicated by the test results in this study, as shown in Figure 5 and Figure 6. In these figures, the measured compressive stresses and strains are normalized by the compressive strength and the strain at the compressive strength, respectively, because the measured compressive strength and the corresponding strain varied depending on the fiber volumetric ratio and fiber aspect ratio.

In order to investigate the effect of test variables on post-peak compressive behavior, one of the three test results extensively measured during large compressive strains was chosen for each test series. The post-peak compressive behavior for some specimens with a relatively low fiber volumetric ratio (V1 series) or with high compressive strength of concrete (H series) could not be measured until the ultimate fracture because the applied load drastically dropped upon failure. As shown in Figure 5, more residual compressive stress after peak stress was observed in the specimens with a larger fiber volumetric ratio. As shown in Figure 6, the specimens containing fibers with a higher aspect ratio showed more ductile post-peak compressive behavior, since a greater transverse confinement effect could be attained by the better pull-out behavior of the steel fibers. This tendency, an effect of the fiber aspect ratio, was investigated through tensile tests, in which the NF3 series generally showed the most desirable performance in terms of tensile behavior [21].

## 4. Model for the Compressive Behavior of SFRC Members

As discussed in the previous section, the compressive behavior of SFRC is significantly affected by the fiber volumetric ratio, fiber aspect ratio, and compressive strength. In this section, the simple formulae for the elastic modulus and the strain at the compressive strength, which represents the pre-peak compressive behavior of SFRC, are derived from the test results. Finally, a model for the compressive behavior of SFRC including the post-peak behavior will be presented with consideration for the effect of steel fibers by employing the fiber reinforcing index, *RI* = *V_f_l_f_*/*d_f_*, which was introduced by previous researchers [11,13,14,15] (Table 1). The models proposed in this paper will be verified through comparison with the test results. The models presented in Table 1 will also be compared with the proposed model as well as the test results.

### 4.1. Strain at the Compressive Strength

**Figure 7:**
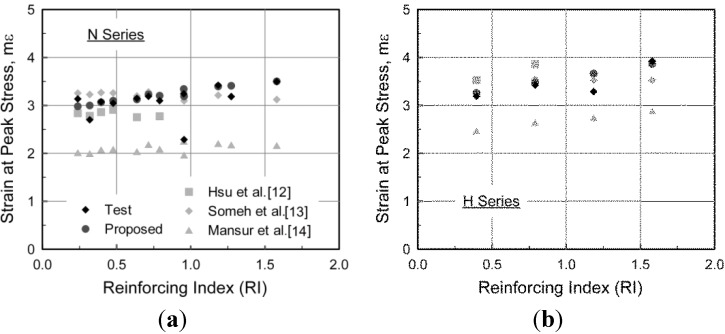
Comparison of the strain at the compressive strength. (**a**) N Series; (**b**) H Series.

Based on the effect of steel fibers for which the strain at the compressive strength is significantly affected by both the fiber volumetric ratio and fiber aspect ratio, the following equation to predict the strain at the compressive strength of SFRC was derived from regression analysis to minimize the square root error corresponding to differences between predictions and test results.

(2) $$\varepsilon_{0}=\left(0.0003V_{f}\frac{l_{f}}{d_{f}}+0.0018\right){f_{c}^{\prime }}^{0.12}=\left(0.0003RI+0.0018\right){f_{c}^{\prime }}^{0.12}$$

where $f_{c}^{\prime }$ is the compressive strength of SFRC. In Figure 7, the strains at the compressive strength measured from the compression test are compared with predictions made using Equation (2) and by other researchers [12,13,14]. Although some researchers [11,15] also proposed their own simple equations to predict the strain at the compressive strength, their models are not included in this comparison because those models are based on the compressive strength of plain concrete. The test results and the model presented in Equation (2) show that the strain at the peak stress gradually increases with increasing fiber reinforcing index (Figure 7). As shown in the figure, Hsu and Hsu [12] predicted the strain at the compressive strength with an acceptable margin, but its application is limited to the specific fiber content outside the common practical range of 1.0%–1.5%, as previously mentioned. Someh and Saeki [13] predicted that the strain at the compressive strength would be nearly constant regardless of the steel fiber content because only the compressive strength of SFRC was taken into account. Mansur *et al.* [14] predicted the increasing tendency of strain at peak stress with an increase in the fiber reinforcing index, but they significantly underestimated the strain at the compressive strength. This result and investigation apply to both N and H series. It can be concluded, therefore, that the strain at the compressive strength can be more reasonably predicted by the model proposed in this paper.

### 4.2. Elastic Modulus

In the same manner as the strain at the compressive strength, the following equation for the elastic modulus was derived from regression analysis:

(3) $$E_{c}=\left(-367V_{f}\frac{l_{f}}{d_{f}}+5520\right){f_{c}^{\prime }}^{0.41}$$

**Figure 8:**
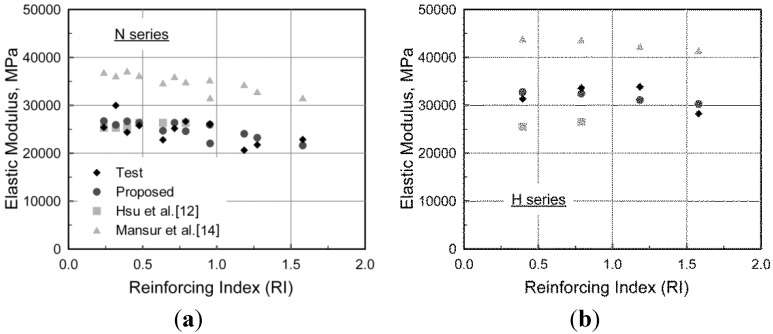
Comparison of the elastic modulus. (**a**) N Series; (**b**) H Series.

In Figure 8, the elastic modulus predicted by the equation was compared with the test results and predictions of previous researchers [12,14]. The model proposed by Ezeldin and Balaguru [11] is not included in the comparison because the compressive strength of plain concrete without steel fibers using the same mixture as SFRC is required to predict the elastic modulus. As presented in the figure, Mansur *et al.* [14] predicted how the elastic modulus would vary, but they generally overestimated the elastic modulus, since the elastic modulus was evaluated from the tangential stiffness at initial loading. This differs from the evaluation procedure used in this paper, in which the secant stiffness between the initial and 40% of the compressive strength was used for the elastic modulus, as presented in ASTM C469 [23]. Although the predictions made by Hsu and Hsu [12] were consistent with the test results for N series, the application of their model is limited to specific fiber levels. On the other hand, extensive testing revealed that Equation (3) accurately predicted the elastic modulus by considering the effect of steel fibers.

### 4.3. Compressive Stress-Strain Relationship

To simulate the stress-strain response of SFRC under compression, the two phases of pre- and post-peak behaviors should be considered separately because no significant lateral crack opening is observed in pre-peak compressive behavior, while the transverse confinement effect of steel fibers to restrain lateral crack opening is considerable in post-peak compressive behavior. As previous research groups’ models summarized in Table 1 are based on the model presented by Carreira and Chu [18], the following equation was employed to represent the compressive behavior of SFRC:

(4) $$f_{c}=f_{c}^{\prime }\left[\frac{A\left(\varepsilon_{c}/\varepsilon_{0}\right)}{A-1+{\left(\varepsilon_{c}/\varepsilon_{0}\right)}^{B}}\right]$$

where *A* and *B* are parameters used to reflect the effect of steel fibers on compressive behavior. For the pre-peak compressive behavior, parameters *A* and *B* in Equation (4) can be calculated by adopting the basic form presented by Carreira and Chu [18] and Mansur *et al.* [14] as follows:

(5) $$A=B=\frac{1}{1-\left(\frac{f_{c}^{\prime }}{\varepsilon_{0}E_{c}}\right)}\text{ for }\varepsilon_{c}/\varepsilon_{0}\leq 1.0$$

where *ε*_0_ and *E_c_* can be calculated from Equations (2) and (3), respectively.

For the post-peak compressive behavior, parameters *A* and *B* have been derived through regression analysis by employing the fiber reinforcing index, $V_{f}l_{f}/d_{f}$.

(6) $$A=1+0.723{\left(V_{f}\frac{l_{f}}{d_{f}}\right)}^{-0.957}\text{ for }\varepsilon_{c}/\varepsilon_{0}>1.0$$

(7) $$B={\left(\frac{f_{c}^{\prime }}{50}\right)}^{0.064}\left[1+0.882{\left(V_{f}\frac{l_{f}}{d_{f}}\right)}^{-0.882}\right]\geq A\text{ in Equation (6) for }\varepsilon_{c}/\varepsilon_{0}>1.0$$

*B* calculated by Equation (7) should not be less than *A* calculated by Equation (6) to prevent the compressive stress in the post-peak behavior from being higher than the compressive strength. This limitation on the parameters can be determined through the derivative of Equation (4) for the post-peak compressive behavior.

**Figure 9:**
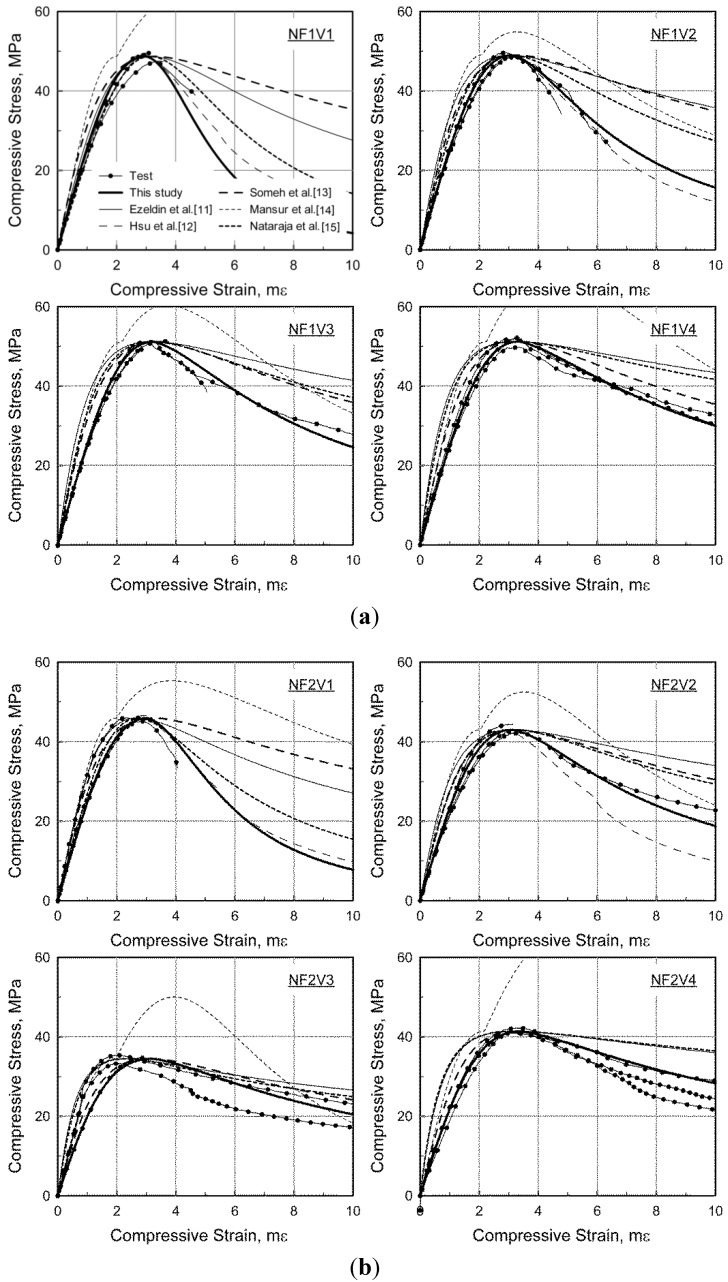

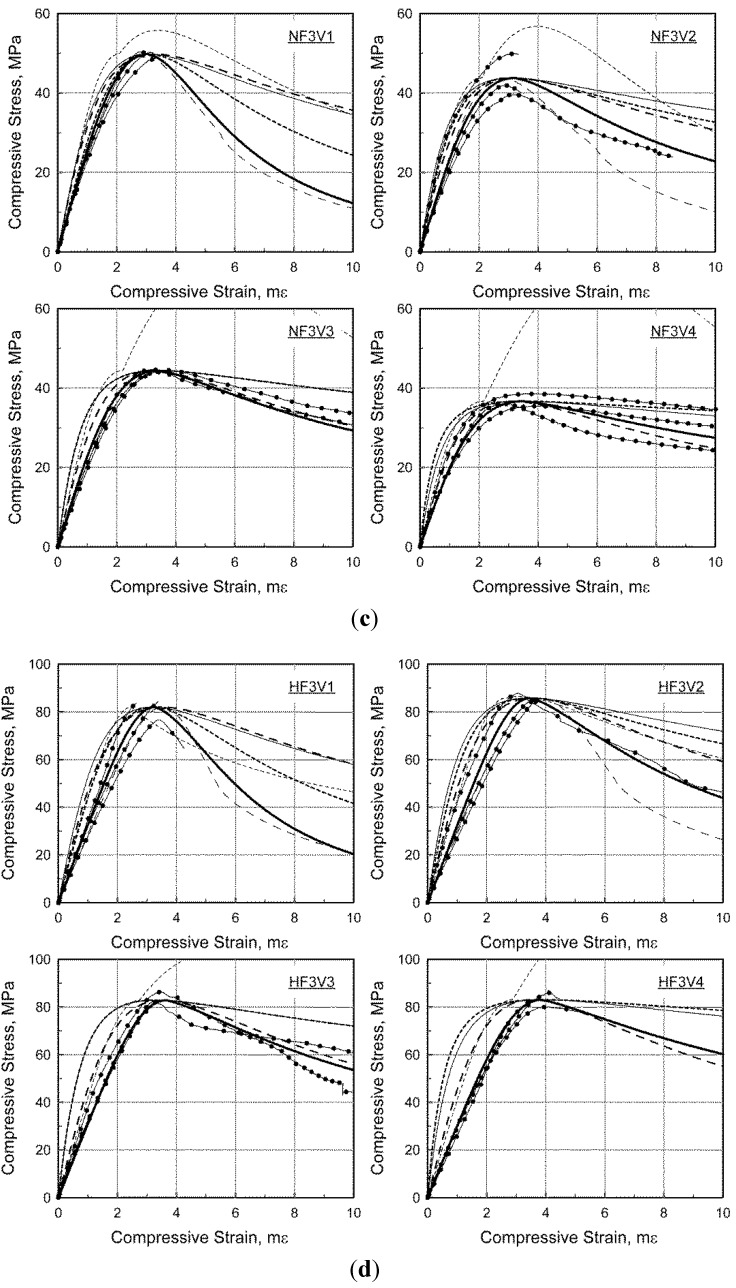
Comparison of the stress-strain response under compression. (**a**) NF1 series; (**b**) NF2 series; (**c**) NF3 series; (**d**) HF3 series.

Figure 9 compares the test results and predictions made by the model proposed in this paper and by other researchers [11,12,13,14,15]. The concrete compressive strengths and corresponding strains of SFRC measured through the tests were used in the model predictions presented by Ezeldin and Balaguru [11] and Nataraja *et al.* [15], who proposed that the compressive strength and the corresponding strains of SFRC be calculated from the compressive strength of specimens without steel fibers under the same mixture design as SFRC. As presented in the figures, although the measured compressive strength and corresponding strain were used, both Ezeldin and Balaguru [11] and Nataraja *et al.* [15] significantly overestimated the residual stress in the post-peak compressive behavior. Moreover, a stiffer compressive response was predicted before the compressive strength was reached. This indicates that the effect of steel fibers on the compressive behavior cannot reasonably be taken into an account in those models, even though the actual compressive strength and corresponding strain are used. This result might due to specimens being small compared to the fiber lengths [11], or a different type of fiber, *i.e.*, crimped fiber being used in their test programs [15].

Hsu and Hsu [12] generally made good predictions of the compressive behavior of the specimens with a low fiber volumetric ratio and fibers with a low aspect ratio (see NF1V1 and NF1V2), but they underestimated the residual stress on the post-peak compressive behavior of the specimens with steel fibers for which the aspect ratio was relatively high (see NF3V1 and NF3V2). In other words, since the model presented by Hsu and Hsu [12] only considers the effect of the fiber volumetric ratio, the effect of the fiber aspect ratio cannot be considered in the prediction of the post-peak compressive behavior of SFRC. Moreover, this model is only applicable to specimens in which the fiber volumetric ratio is not greater than 1.0%.

Someh and Saeki [13] successfully predicted only the compressive behavior of specimens with a high fiber volumetric ratio and steel fibers with a high aspect ratio (see NF3V3, NF3V4 and HF3V3). For other specimens, the residual stress in the post-peak compressive behavior was significantly overestimated. The prediction of Mansur *et al.* [14] was not applicable since the compressive stress in the post-peak behavior was higher than the compressive strength when *k*_1_ was larger than *k*_2_ in the model summarized in Table 1. On the other hand, the compressive behavior predicted by Equations (4)–(7) proposed in this paper showed good agreement with the test results for various concrete compressive strengths and fiber contents.

## 5. Conclusions

Although many researchers proposed several models from their own test results in order to quantitatively evaluate the compressive behavior of SFRC, the appropriateness of the previous models is still questionable since the variables were too limited to extensively reflect the effect of steel fibers, or since the test specimens for the investigation were relatively small compared to the fiber length. The compressive behavior may have been different from those in real structures because of different fiber distributions.

In this paper, an experimental program was implemented to investigate the compressive behavior of SFRC with end-hooked steel fibers. The variables of the test program were concrete compressive strength, fiber volumetric ratio, and fiber aspect ratio. To minimize the effect of member size on fiber distribution, large cylindrical specimens were prepared and tested. Comparison between the predictions of other researchers’ models and the test results revealed that no previous model could acceptably reflect the effect of end-hooked steel fibers on the compressive behavior.

In order to better represent the pre-peak compressive behavior of SFRC, simple formulae to predict the strain at the compressive strength and the elastic modulus were derived from the test results. In addition, by modifying the coefficient *β*, a simple model to predict the compressive behavior of SFRC was proposed, with consideration for the effect of steel fibers. The compressive behavior of SFRC with end-hooked steel fibers can be reasonably predicted using the proposed formulae and model. The proposed model may prove useful for predicting the structural behavior of SFRC members or structures with end-hooked steel fibers. Further study is required to understand the effect of other fiber types, such as straight steel fibers and synthetic fibers. In addition, through more investigation for the effect of SFRC member size on the compressive behavior, the proposed model can be applied to SFRC members with various dimensions.
